# Predictors of infarction and outcomes in capsular warning syndrome: a retrospective observational study

**DOI:** 10.3389/fneur.2025.1617623

**Published:** 2025-07-28

**Authors:** Rui-xi Guo, Tao Wang

**Affiliations:** ^1^The Affiliated Hospital of Yan'an University, Yan'an, Shaanxi, China; ^2^Department of Neurology, Yan'an People's Hospital, Yan'an, China

**Keywords:** capsular warning syndrome, transient ischemic attack (TIA), LASSO regression, infarction, tirofiban

## Abstract

**Background:**

Capsular warning syndrome (CWS), a rare subtype of transient ischemic attack, is associated with a high risk of progression to acute cerebral infarction. However, predictive factors for infarction and determinants of functional outcome remain inadequately defined.

**Methods:**

In this retrospective study, we analyzed 89 CWS patients admitted between January 2021 and December 2024. Clinical, laboratory, imaging, and treatment data were collected. Patients were stratified into infarcted and non-infarcted groups based on DWI findings. Infarcted patients were followed for 90 days, with outcomes assessed using the modified Rankin Scale (mRS). LASSO regression was used for variable selection, followed by multivariable logistic regression to identify independent predictors of infarction and favorable 90-day outcomes (mRS 0–2).

**Results:**

Of 89 patients, 57 (64%) experienced infarctions. Independent predictors included elevated platelet count (OR = 1.02, 95% CI: 1.01–1.04, *p* = 0.002), higher NIHSS score (OR = 1.96, 95% CI: 1.25–3.55, *p* = 0.011), higher ABCD2 score (OR = 1.86, 95% CI: 1.04–3.60, *p* = 0.047), intracranial atherosclerosis (OR = 10.5, 95% CI: 1.54–99.0, *p* = 0.024), and male sex (OR = 5.57, 95% CI: 1.32–27.9, *p* = 0.024). Among 57 infarcted patients, tirofiban infusion was the only independent predictor of favorable outcome (OR = 0.01, CI: 0.00–0.07, *p* < 0.001).

**Conclusion:**

In CWS, infarction risk is independently associated with platelet count, clinical severity, vascular pathology, and sex. Tirofiban may improve short-term outcomes in infarcted patients. Prospective multicenter studies are needed to validate these findings.

## Graphical abstract

Risk stratification and therapeutic modeling in capsular warning syndrome.
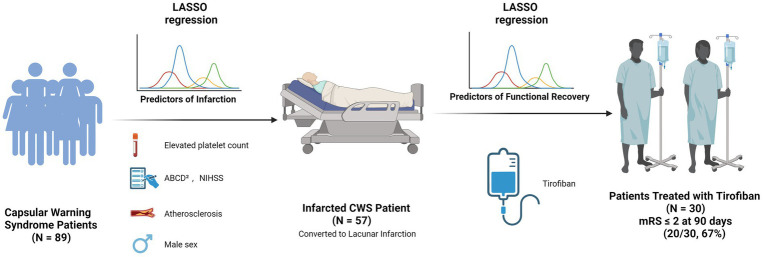

## Introduction

Capsular warning syndrome (CWS) is an uncommon but distinctive clinical manifestation of acute ischemic cerebrovascular disease, currently recognized as a specific subtype of transient ischemic attack (TIA) (1). First characterized by Donnan in 1993, CWS is clinically defined by at least three stereotyped, transient, and completely reversible episodes of unilateral motor or sensory impairment occurring within a 24-h period. These episodes must involve at least two regions among the face, arm, or leg, without associated cortical dysfunctions such as aphasia, apraxia, agnosia, or cognitive impairment (2). CWS is most commonly associated with lacunar infarctions, which result from the occlusion of small penetrating arteries. Furthermore, TIA have been observed in approximately 10% of patients prior to the onset of lacunar infarction, suggesting a prodromal phase in some cases (3, 4). Recent advancements in neuroimaging have clarified that lesions underlying CWS are not restricted solely to the internal capsule, as initially postulated, but may extend to other deep cerebral structures, including the pons, thalamus, centrum semiovale, and corpus callosum (5, 6). Additionally, the temporal definition of CWS has evolved, with recent studies proposing expanded diagnostic windows of 48 h, 72 h, or even up to 7 days, leading to broader and more inclusive diagnostic criteria (7–9).

Despite clinical parallels with conventional TIA, CWS is associated with a significantly increased risk of progression to ischemic stroke. The 90-day stroke risk among general TIA patients is approximately 1.4% (10), whereas the reported rate of infarction among CWS patients ranges from 71.2 to 80.55% (11, 12). However, comprehensive investigations into independent risk factors specifically predicting infarction in CWS remain limited. Current literature predominantly addresses conventional vascular risk factors such as hypertension, atherosclerosis, and hyperhomocysteinemia (13). Currently, clinical management of CWS often mirrors that of TIA or acute ischemic stroke, involving antiplatelet therapy and intravenous thrombolysis (14). Among antiplatelet options, tirofiban is a fast-acting, reversible glycoprotein IIb/IIIa antagonist that inhibits the final step of platelet aggregation. Its safety and potential benefits in acute ischemic stroke have been supported by several studies (15, 16). Considering the potential contribution of platelet hyperreactivity and small-vessel pathology in CWS, tirofiban represents a biologically rational candidate for mitigating infarct development. Nevertheless, the efficacy and safety of these strategies in CWS remain uncertain due to the lack of high-quality, CWS-specific evidence. Heterogeneous designs and limited sample sizes further contribute to inconsistent conclusions and the absence of standardized treatment guidelines.

This study aims to review the clinical and imaging characteristics of CWS, identify predictors of infarction and poor prognosis, and evaluate the impact of different therapeutic strategies on clinical outcomes. By elucidating risk profiles and treatment effects in this unique population, we hope to inform earlier recognition and tailored interventions for patients at high risk of progression.

## Materials and methods

### Study design and population

This retrospective observational study included consecutive patients admitted to the Department of Neurology at Yan’an University Affiliated Cardiovascular and Cerebrovascular Hospital between January 2021 and December 2024 for acute ischemic stroke. All patients met the clinical criteria for CWS and were managed according to standard institutional protocols. The study was approved by the hospital’s institutional ethics committee (Approval No. L2020184), and all patient data were anonymized for confidentiality.

### Inclusion criteria

1. Age ≥18 years.
2. Clinical diagnosis of CWS, defined as ≥3 stereotyped, transient, and fully reversible motor or sensory episodes within 24 h, involving at least two of the face, arm, or leg, without cortical signs (e.g., aphasia, neglect).
3. Time from symptom onset to hospital admission ≤24 h.

### Exclusion criteria

1. Altered level of consciousness at admission.
2. Neuroimaging or medical history indicating alternative cerebrovascular or intracranial diseases (e.g., intracerebral hemorrhage, chronic subdural hematoma, neoplasm, subarachnoid hemorrhage, arteriovenous malformation, or aneurysm).
3. Stroke mimics (e.g., postictal paresis, functional neurological disorder, migraine aura, neuromyelitis optica spectrum disorder).
4. Severe systemic illness precluding standard stroke care (e.g., end-stage renal disease, decompensated heart failure).
5. Use of anticoagulation with high bleeding risk, known coagulopathy, or platelet count <100 × 10^9^/L.

### Definitions of comorbidities

1. Hypertension: Documented history or use of antihypertensives, or admission blood pressure ≥130/90 mmHg.
2. Diabetes Mellitus: Documented history or use of antidiabetic drugs, or fasting glucose ≥7.0 mmol/L, or HbA1c ≥ 6.5%.
3. Dyslipidemia: Documented diagnosis or lipid-lowering therapy, or LDL-C ≥ 3.4 mmol/L, total cholesterol ≥5.2 mmol/L, or triglycerides ≥1.7 mmol/L.
4. Hyperhomocysteinemia: Known diagnosis or fasting plasma homocysteine >15 μmol/L.
5. Atherosclerosis: Based on prior history or neurovascular imaging showing vessel wall thickening, plaque, or stenosis.
6. Prior stroke/TIA, demyelinating lesions, smoking, and alcohol use were determined from medical records and patient interviews.

### Data collection

1. Demographics: age, sex.
2. Clinical presentation: frequency and duration of attacks.
3. Medical history and comorbidities (as defined above).
4. Laboratory data: CBC, coagulation, glucose, lipid panel, homocysteine.
5. Imaging: DWI for infarction status and lesion localization; CTA/MRA for atherosclerosis. (Emergency repeat DWI imaging was conducted during hospitalization upon neurological deterioration lasting over 1 hour or an NIHSS increase of ≥2 points, guiding subsequent therapeutic decisions).
6. Stroke scales: ABCD^2^ and NIHSS at presentation; modified Rankin Scale (mRS) at 90 days.
7. Treatment: rt-PA, intravenous tirofiban, and oral antiplatelet therapy.

### Treatment protocol

All patients received standard oral antiplatelet therapy upon admission. Tirofiban therapy was initiated once patients exhibited persistent, unrelieved CWS symptoms—defined as a neurological deficit lasting > 60 min *or* recurrent attacks without full recovery within 1 h—despite standard dual oral antiplatelet therapy, irrespective of DWI findings. The standardized tirofiban protocol was:

1. Loading: 0.4 μg·kg^−1^·min^−1^ for 30 min.
2. Maintenance: 0.1 μg·kg^−1^·min^−1^ for 24 h (halved if eGFR <30 mL·min^−1^).
3. Antiplatelet overlap: Aspirin (100 mg) + clopidogrel (75 mg) started 4 h before infusion discontinuation.

### Study design and outcomes

1. Phase 1: All patients clinically diagnosed with CWS were included and grouped based on DWI results (positive vs. negative). The primary outcome was DWI-confirmed infarction.
2. Phase 2: Patients with confirmed infarction (DWI-positive) were followed for 90 days. The primary outcome was functional independence (mRS 0–2).

### Statistical analysis

Comparisons were made using chi-square or Fisher’s exact test for categorical variables, and t-test or Mann–Whitney U test for continuous variables, as appropriate. LASSO regression (using the 1-SE rule) was used for variable selection, followed by multivariable logistic regression. Odds ratios (ORs) with 95% confidence intervals (CIs) were reported. A *p*-value <0.05 was considered statistically significant. To evaluate the predictive performance of the final multivariate logistic regression model, we performed receiver operating characteristic (ROC) curve analysis. Analyses were performed using R software (version 4.2.2).

## Results

### Baseline characteristics

A total of 89 patients diagnosed with CWS were included. Of these, 64 (72%) were male and 25 (28%) females, with a mean age of 58 ± 11 years. The most common comorbidities included hypertension (58%), dyslipidemia (56%), prior stroke (27%), and diabetes (18%). Additionally, 47% had a history of smoking and 19% reported alcohol use.

**Figure 1:**
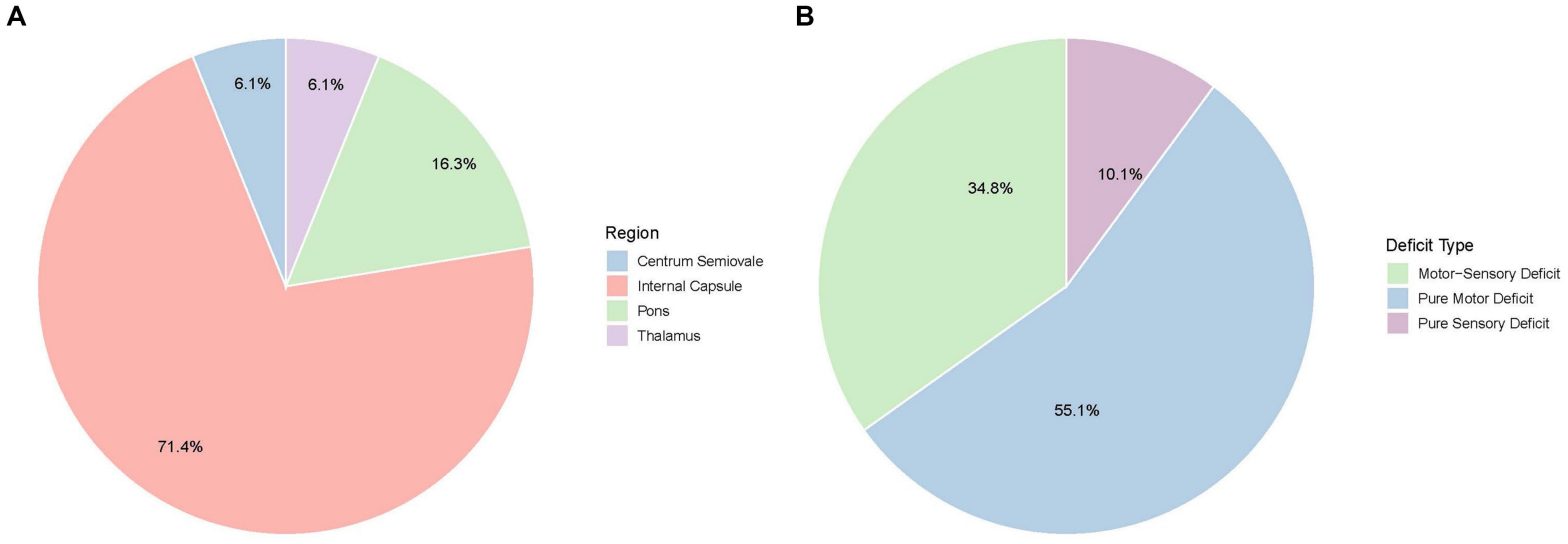
Clinical and imaging characteristics of capsular warning syndrome. **(A)** Distribution of present symptoms among patients with CWS. **(B)** Anatomic locations of acute infarcts detected by diffusion-weighted imaging (DWI).

All patients presented with recurrent, transient neurological deficits. The median number of episodes per patient was 4 (range: 3–20). Symptom patterns were classified as purely motor in 49 patients (55.1%), combined motor and sensory in 31 (34.8%), and purely sensory in 9 (10.1%) (Figure 1A). Median ABCD2 and NIHSS scores at presentation were 3 and 4, respectively. The median duration of each episode was 10 min.

### Imaging findings

DWI revealed acute infarctions in 57 patients (64%). The most commonly affected region was the internal capsule (42 patients, 73.7%), particularly the posterior limb. Additional infarcts were observed in the pons (15.8%), thalamus (5.3%), and centrum semiovale (5.3%) (Figure 1B). Intracranial atherosclerosis was identified in 73 patients (82%) via vascular imaging, though only 5 (5.6%) had significant large artery stenosis or occlusion.

### Risk factors for infarction

**Figure 2:**
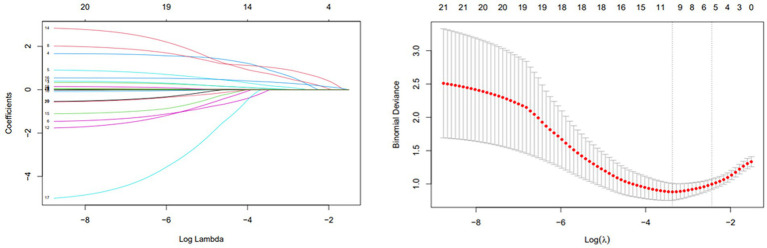
LASSO regression for selecting predictors of acute infarction in CWS.

Patients were categorized into infarcted (*n* = 57) and non-infarcted (*n* = 32) groups based on DWI findings. Clinical and laboratory comparisons were made between the two groups (Table 1). LASSO regression (Figure 2) followed by multivariable logistic regression revealed that elevated platelet count (OR = 1.02, 95% CI: 1.01–1.04, *p* = 0.002), higher NIHSS score (OR = 1.96, 95% CI: 1.25–3.55, *p* = 0.011), higher ABCD2 score (OR = 1.86, 95% CI: 1.04–3.60, *p* = 0.047),presence of intracranial atherosclerosis (OR = 10.5, 95% CI: 1.54–99.0, *p* = 0.024), and male sex (OR = 5.57, 95% CI: 1.32–27.9, *p* = 0.024) were independent predictors of infarction (Table 2).

**Table 1:** Baseline clinical and treatment characteristics of CWS patients by infarction status.

Characteristic	Overall *N* = 89	Non-Infarct *N* = 32	Infarct *N* = 57	Test Statistic^2^	*p*-value
Demographics^1^					
Age	58 (11)	60 (10)	58 (11)	*t* = 0.822	0.4
Sex				*χ*^2^=13.628	**<0.001**
Female	25 (28%)	17 (53%)	8 (14%)		
Male	64 (72%)	15 (47%)	49 (86%)		
Laboratory-based data^1^					
APTT (s)	25.82 (2.40)	26.21 (2.44)	25.60 (2.37)	*t* = 1.139	0.3
PLT (×10^9^/L)	252 (66)	225 (54)	267 (67)	*t* = −3.188	**0.002**
Glucose (mmol/L)	5.50 (4.90,6.70)	5.33 (4.90,6.15)	5.60 (4.80,6.71)	U Test	0.4
TG (mmol/L)	1.43 (1.02,2.01)	1.47 (1.09,1.99)	1.33 (0.99,2.01)	U Test	0.5
PCT (ng/mL)	0.25 (0.21,0.29)	0.23 (0.21,0.26)	0.26 (0.21,0.29)	U Test	0.2
PT (s)	11.00 (10.60,11.40)	11.05 (10.80,11.25)	11.00 (10.50,11.50)	U Test	0.8
Clinical presentation^1^					
Attack frequency	4.00 (3.00,5.00)	3.00 (3.00,4.50)	4.00 (4.00,5.00)	U Test	0.088
Attack duration (min)	10 (6,28)	10 (5,15)	10 (10,28)	U Test	0.057
NIHSS	4.00 (3.00,6.00)	2.50 (2.00,4.00)	5.00 (4.00,7.00)	U Test	**<0.001**
ABCD2	3.00 (3.00,4.00)	3.00 (2.00,4.00)	4.00 (3.00,4.00)	U Test	**<0.001**
Comorbidities, *n* (%)^1^					
Diabetes	16 (18%)	6 (19%)	10 (18%)	*χ*^2^=0	>0.9
Hypertension	52 (58%)	20 (63%)	32 (56%)	*χ*^2^=0.13	0.7
Hyperlipidemia	50 (56%)	18 (56%)	32 (56%)	*χ*^2^=0	>0.9
Smoke	42 (47%)	8 (25%)	34 (60%)	*χ*^2^=8.532	**0.003**
Atherosclerosis	73 (82%)	22 (69%)	51 (89%)	*χ*^2^=4.646	**0.031**
Demyelinating lesions	48 (54%)	15 (47%)	33 (58%)	*χ*^2^=0.607	0.4
Stroke	24 (27%)	10 (31%)	14 (25%)	*χ*^2^=0.188	0.7
Infarction	40 (45%)	13 (41%)	27 (47%)	*χ*^2^=0.153	0.7
HCY	41 (46%)	7 (22%)	34 (60%)	*χ*^2^=10.299	**0.001**
Drinking	17 (19%)	2 (6.3%)	15 (26%)	Fisher Exact Test	**0.025**
Treatment information^1^					
rt-PA	35 (39%)	13 (41%)	22 (39%)	*χ*^2^=0.02	0.9
Tirofiban	42 (47.2%)	12 (37.5%)	30 (52.6%)	*χ*^2^=1.32	0.25
Antiplatelet	89 (100%)	32 (36%)	57 (64%)	*χ*^2^=0.01	0.9

^1^Mean (SD); *n* (%).

^2^Welch two sample t-test; Wilcoxon rank sum test; Pearson’s Chi-squared test; Fisher’s exact test.

Bold values indicate statistical significance at *p* < 0.05.

**Table 2:** Multivariate logistic regression of LASSO-selected risk factors for acute infarction in CWS.

Characteristic^1^	OR	95% CI^1^	*p*-value^1^
PLT	1.02	1.01, 1.04	0.002
NIHSS	1.96	1.25,3.55	0.011
ABCD2	1.86	1.04, 3.60	0.047
Atherosclerosis			
No	1.00	1.00, 1.00	—
Yes	10.5	1.54, 99.0	0.024
HCY			
No	1.00	1.00, 1.00	—
Yes	2.52	0.67, 9.91	0.2
Sex			
Female	1.00	1.00, 1.00	—
Male	5.57	1.32, 27.9	0.024

^1^Logistic regression.

CI = confidence interval, OR = odds ratio.

**Figure 3:**
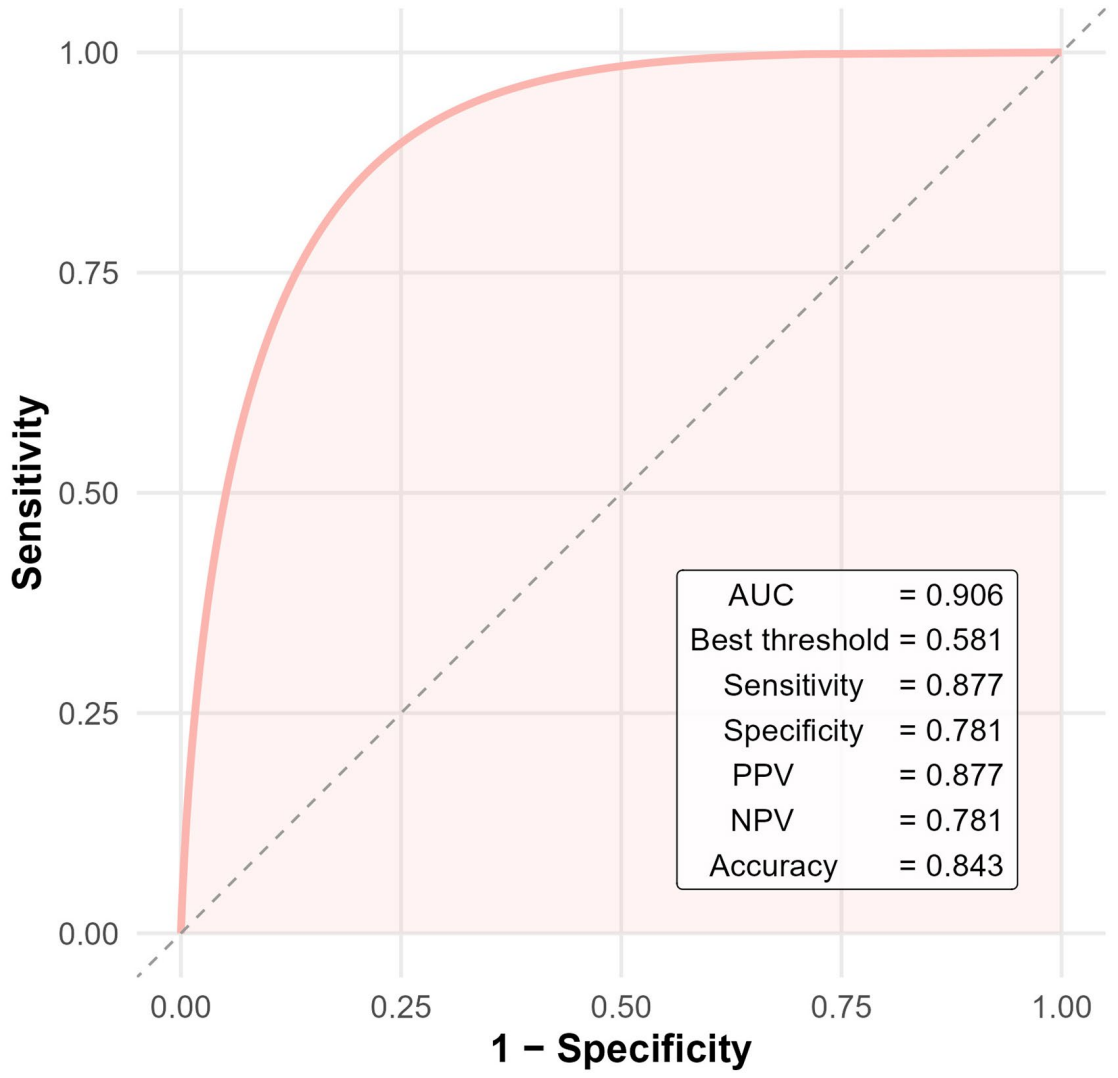
ROC curve of the multivariate logistic regression model for predicting cerebral infarction in patients with CWS.

To further evaluate the diagnostic performance of the multivariate model, receiver operating characteristic (ROC) analysis was conducted. The model yielded an area under the curve (AUC) of 0.906, indicating excellent discrimination. At the optimal cutoff value of 0.581 (determined by the Youden index), the model demonstrated a sensitivity of 87.7%, specificity of 78.1%, positive predictive value (PPV) of 87.7%, negative predictive value (NPV) of 78.1%, and overall diagnostic accuracy of 84.3% (Figure 3).

### Predictors of 90-day outcome among infarcted patients

**Figure 4:**
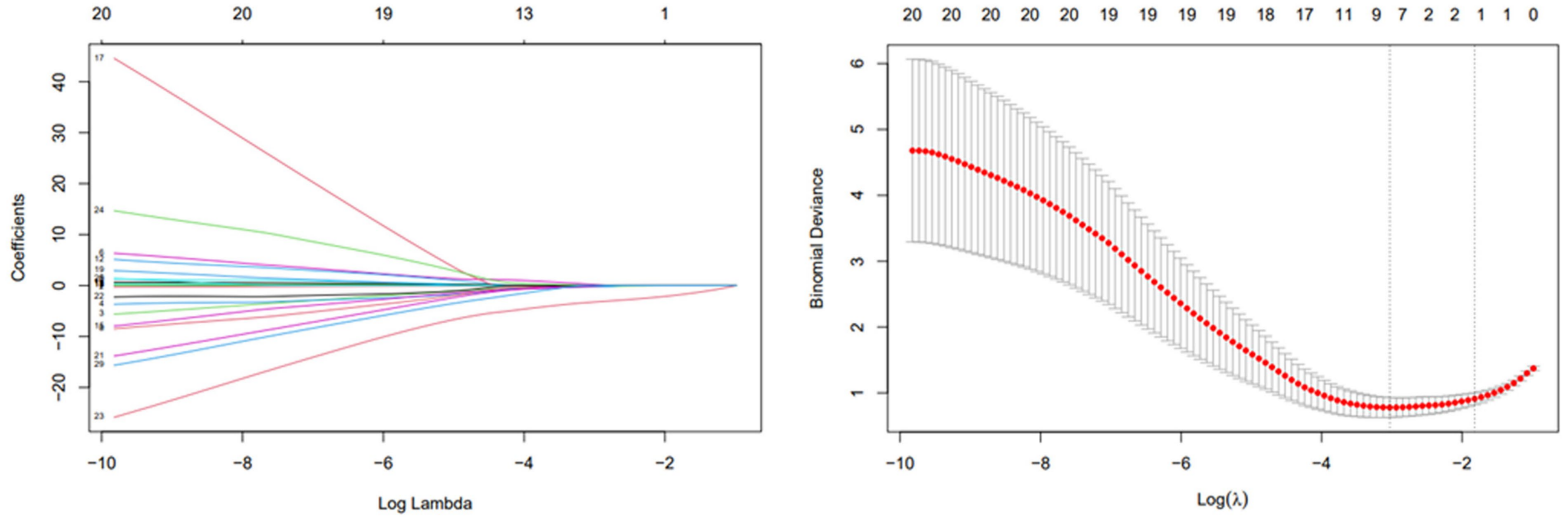
LASSO regression for identifying prognostic factors in infarcted CWS patients.

Of the 57 patients with DWI-confirmed infarction, 30 (53%) met escalation criteria and received an intravenous tirofiban infusion, whereas the remaining 25 (44%) were treated with dual oral antiplatelet therapy alone. At the 90-day evaluation, 31patients (54.3%) achieved functional independence (mRS 0–2), while 26 patients (45.7%) had an unfavorable outcome (mRS > 2). Favorable outcome occurred in 20 of 30 patients who received tirofiban (65%) and in 10 of 25 patients managed with antiplatelet therapy alone (40%). Baseline clinical and treatment characteristics stratified by outcome are summarized in Table 3. LASSO variable selection (Figure 4) followed by multivariable logistic regression identified tirofiban use as the only independent predictor of functional independence at 90 days OR = 0.01, CI: 0.00–0.07, *p* < 0.001 (Table 4).

**Table 3:** Baseline and treatment characteristics of infarcted CWS patients by 90-day outcome.

Characteristic	Overall *N* = 57	mRS (0–2) *N* = 31	mRS (>2) *N* = 26	Test statistic^2^	*p*-value^2^
Demographics^1^					
Age	58 (11)	58 (12)	57 (10)	*t* = 0.152	0.9
Sex				Fisher Exact Test	0.2
Female	8 (14%)	2 (6.5%)	6 (23%)		
Male	49 (86%)	29 (94%)	20 (77%)		
Laboratory-based data^1^					
APTT (s)	25.60 (2.37)	25.60 (2.28)	25.60 (2.51)	*t* = 0.005	>0.9
PLT (×10^9^/L)	272 (55)	258 (57)	289 (48)	*t* = −2.203	**0.032**
Glucose (mmol/L)	5.60 (4.80, 6.71)	5.80 (4.80, 7.20)	5.55 (4.80, 6.71)	U Test	0.9
TG (mmol/L)	1.33 (0.99, 2.01)	1.32 (0.99, 2.10)	1.39 (1.12, 2.01)	U Test	>0.9
PCT (ng/mL)	0.26 (0.21, 0.29)	0.26 (0.22, 0.30)	0.26 (0.20, 0.28)	U Test	0.2
PT (s)	11.00 (10.50, 11.50)	11.00 (10.40, 11.40)	11.15 (10.60, 11.70)	U Test	0.6
Clinical presentation^1^					
Attack frequency	4.00 (4.00, 5.00)	4.00 (4.00, 6.00)	4.00 (3.00, 5.00)	U Test	0.5
Attack duration(min)	10 (10, 28)	15 (10, 30)	10 (5, 25)	U Test	0.2
ABCD2	4.00 (3.00, 4.00)	4.00 (3.00, 4.00)	3.00 (3.00, 4.00)	U Test	0.5
NIHSS	4.00 (3.00, 6.00)	3.50 (3.00, 4.00)	5.00 (4.00, 7.00)	U Test	0.2
Comorbidities, *n* (%)^1^					
Hypertension	32 (56%)	17 (55%)	15 (58%)	*χ*^2^=0	>0.9
Diabetes	10 (18%)	6 (19%)	4 (15%)	Fisher Exact Test	>0.9
Hyperlipidemia	32 (56%)	16 (52%)	16 (62%)	*χ*^2^=0.234	0.6
Smoke	34 (60%)	20 (65%)	14 (54%)	*χ*^2^=0.299	0.6
Stroke	14 (25%)	9 (29%)	5 (19%)	*χ*^2^=0.3	0.6
Infarction	27 (47%)	14 (45%)	13 (50%)	*χ*^2^=0.01	>0.9
HCY	34 (60%)	19 (61%)	15 (58%)	*χ*^2^=0	>0.9
Atherosclerosis	51 (89%)	28 (90%)	23 (88%)	Fisher Exact Test	>0.9
Demyelination	33 (58%)	20 (65%)	13 (50%)	Fisher Exact Test	0.3
Treatment information^1^					
Antiplatelet only	25 (44%)	10 (32%)	15 (58%)	χ^2^ = 5.12	0.054
Tirofiban	30 (53%)	20 (65%)	10 (38%)	Fisher Exact Test	**0.012**
rt-PA	2 (3%)	1 (3%)	1 (4%)	Fisher Exact Test	0.9

^1^Mean (SD); *n* (%).

^2^Welch two sample t-test; Wilcoxon rank sum test; Pearson’s Chi-squared test; Fisher’s exact test.

Bold values indicate statistical significance at *p* < 0.05.

**Table 4:** Multivariate logistic regression of LASSO-selected predictors for favorable 90-day outcome in infarcted CWS patients.

Characteristic^1^	OR	95% CI^1^	*p*-value^1^
Attack duration (min)	0.96	0.90, 1.00	0.088
Attack frequency	0.47	0.15, 1.89	0.12
Tirofiban			
No	1.00	1.00, 1.00	—
Yes	0.01	0.00, 0.07	<0.001
Antiplatelet			
No	1.00	1.00, 1.00	—
Yes	0.69	0.09, 4.98	0.7

^1^Logistic regression.

CI = confidence interval, OR = odds ratio.

## Discussion

Our study set out to clarify two unresolved questions in CWS: (i) which bedside or laboratory variables truly forecast progression to infarction, and (ii) whether intensive, mechanism-directed antiplatelet therapy can improve functional recovery once infarction occurs. By analyzing 89 consecutive patients we show, first, that a composite profile of platelet hyperreactivity (elevated platelet count), early clinical severity (higher NIHSS and ABCD^2^ scores), intracranial atherosclerosis, and male sex independently signals imminent infarction, and second, that early, weight-adjusted tirofiban infusion is associated with a three- to four-fold increase in 90-day independence relative to best-medical therapy alone.

Even modest increases within the high-normal platelet range intensified the probability of permanent infarction (17). Platelets with greater surface density not only raise the encounter rate between GP IIb/IIIa receptors and fibrinogen but also shed pro-coagulant macrovesicles that accelerate thrombin generation (18, 19). Male sex retained significance after multivariable adjustment; testosterone is known to up-regulate platelet thromboxane A₂ receptor density and augment aggregation (20) Intracranial atherosclerosis predicted infarction despite minimal large-artery stenosis in most patients, underscoring the “branch-occlusive” model in which ostial plaques obstruct perforating arteries or create platelet-rich micro emboli (21). Chronic hypertension, smoking, dyslipidemia and hyperhomocysteinemia—present in >70% of our cohort—promote lipohyalinosis, endothelial nitric-oxide depletion and basement-membrane thickening, further narrowing the perforator lumen and predisposing to flow arrest (22, 23).

Tirofiban is a short-acting, reversible glycoprotein IIb/IIIa antagonist that blocks the final common pathway of platelet aggregation without the immunogenicity of abciximab. Experimental models confirm that perforator-type infarcts are exquisitely platelet-dependent and respond poorly to P2Y12 inhibition alone (24). The SaTIS trial established safety in moderate ischemic stroke (25), and successive randomized data have documented functional benefits in selected populations—large-artery atherosclerosis (26), non-occlusive strokes, and, most recently, in combination with intravenous thrombolysis (27). Our cohort complements these trials by focusing on small-vessel-dominant pathology, a context in which platelet-rich thrombi and endothelial plugging are central. The magnitude of benefit we observed is concordant with the pooled effect of four randomized controlled trials in non-repercussed stroke (28). Nevertheless, it contrasts with a neutral signal in a prior open-label CWS series (29), possibly because our protocol mandated (i) early initiation after first sustained deficit and (ii) overlap with dual oral antiplatelets to prevent rebound aggregation. These findings are consistent with recent evidence from a multicenter cohort study, which demonstrated that tirofiban administration improved outcomes in patients with large artery atherosclerosis who achieved complete reperfusion and were presented with high NIHSS scores (30). This further supports the potential utility of tirofiban in patients with CWS, especially those with underlying atherosclerotic pathology and severe initial neurological deficits.

Our conclusions must be interpreted in light of several constraints. First, the retrospective design invites selection bias, particularly in treatment allocation—patients receiving tirofiban had more severe intrahospital fluctuations, which could lead to confounding by indication despite multivariate adjustment. Second, sample size is modest, limiting power for subgroup analyses (e.g., sex-specific efficacy) and inflating confidence intervals around some estimates. Third, lack of randomization and blinding leaves room for unmeasured confounders, including clinician preference and temporal improvements in ancillary care. Fourth, precise symptom onset-to-admission intervals were not consistently documented. However, all included patients were admitted within 24 h from symptom onset. Finally, generalizability is restricted: our hospital is a tertiary referral center in north-west China with a high background prevalence of intracranial atherosclerosis; centers with different risk profiles or practice patterns may not reproduce identical effect sizes.

Taken together, our data support a two-step management algorithm: bedside triage using the platelet-NIHSS-ABCD^2^ composite to identify high-risk patients, followed by early tirofiban infusion in those who convert to infarction despite standard therapy. Prospective, multicenter randomized trials with stratification by platelet function and imaging-defined perforator disease are urgently required. Incorporation of blood biomarkers of endothelial dysfunction and high-resolution vessel-wall MRI could further unravel the microvascular mechanisms precipitating CWS and refine candidate selection for intensive antiplatelet therapy.

## Conclusion

In this single-center cohort, elevated platelet count, high initial clinical scores, intracranial atherosclerosis, and male sex independently predicted infarction in CWS, while low-dose tirofiban was the only modifiable factor associated with superior 90-day functional outcomes. These findings strengthen the biological rationale for early, mechanism-specific platelet inhibition in high-risk CWS and lay the groundwork for definitive randomized evaluation.

## Data Availability

The raw data supporting the conclusions of this article will be made available by the authors, without undue reservation.
